# Influence of Fluorine Doping of Activated Carbon Fibers on Their Water Vapor Adsorption Characteristics

**DOI:** 10.3389/fchem.2020.593756

**Published:** 2021-01-06

**Authors:** Leticia F. Velasco, Kyung Hoon Kim, Young-Seak Lee, Peter Lodewyckx

**Affiliations:** ^1^Department of Chemistry, Royal Military Academy, Brussels, Belgium; ^2^Department of Chemical Engineering and Applied Chemistry, Chungnam National University, Daejeon, South Korea

**Keywords:** water sorption, fluorine doping, activated carbon fibers, surface chemistry, hydrophilicity

## Abstract

The characterization of fluorinated carbon fibers by water sorption has been broadly investigated in this work. In brief, a pitch-based activated carbon fiber (ACF) was submitted to a fluorination process under different conditions of partial pressure (F_2_:N_2_ ratio) and temperature. This led to samples with varied fluorine content and C-F type bonding. The effect of the fluorination treatment on the textural properties of the ACF was studied by means of nitrogen and carbon dioxide adsorption at −196 and 0°C, respectively, while the changes induced in the surface chemistry of the materials were analyzed by XPS. Also, the affinity and stability of the materials toward water was evaluated by single and cycling isotherms. The obtained results show that a mild fluorination not only can preserve most of the textural properties of the parent ACF, but enhance the water uptake at the first stages of the water sorption process, together with a shift in the upswing of the water isotherms toward lower relative humidities. This indicates that fluorination under certain conditions can actually enhance the surface hydrophilicity of carbon materials with specific properties. On the contrary, higher partial pressures led to highly fluorinated fibers with lower porosity and more hydrophobic character. Moreover, they presented a lower chemical stability as demonstrated by a change in the shape of the water isotherms after two consecutive measurements. The kinetics of water sorption in the ACFs provided further insights into the different sorption phenomena involved. Hence, water sorption can definitely help to tailor the water affinity, stability and performance of fluorinated porous carbon materials under humid conditions.

## Introduction

The mechanisms of water sorption in carbon pores have been the subject of extensive study during the last years (Liu et al., 2017). Hence, as the process gets better understood, the use of water isotherms for the characterization of porous carbon materials is gaining interest. This is mainly due to its advantages over other adsorbates: it can be performed at room temperature, water has a very small kinetic diameter (0.28 nm) which allows it to enter pores even smaller than those accessible to CO_2_ (Lodewyckx, 2010) and it gives information about both the textural and surface properties of the material. In spite of this, the potential of this technique is still far from being fully explored.

It is well-established that water sorption is sensitive to surface chemistry (Bandosz et al., 1996; Thommes et al., 2011; Tóth and Lászlo, 2012; Lodewyckx et al., 2013; Nguyen et al., 2014). More specifically, it is assumed that water adsorption in carbon nanomaterials first proceeds through the clustering of water molecules around primary adsorption sites (functional surface groups). Thus, the overall shape of the water isotherm, and more particularly the first part, can give important information about the surface composition, which is a crucial feature for the performance of the material under realistic conditions of humidity.

The doping of carbon materials with heteroelements is known to be an effective approach to tailor their hydrophilic/hydrophobic character and consequently their water adsorption behavior (Lee et al., 2007; Kumar et al., 2016). In this regard, fluorinated porous carbonaceous materials are receiving increasing attention due to their promising performance in several fields such as gas adsorption, gas sensors or energy storage (Touhara and Okino, 2000; Park et al., 2016; Kim et al., 2018; Wang et al., 2020) and they can even be considered as a new class of materials (Matei Ghimbeu et al., 2015; Shahtalebi et al., 2016). In the specific case of activated carbon fibers, fluorination has proven effective for improving their electrochemical properties (Shao et al., 2016) as well as the methane storage capacity (Im et al., 2009), CO_2_ adsorption (Sugiyama and Hattori, 2020), and even for biomedical applications (Sun et al., 2017). Nevertheless, the mechanisms of the fluorination process and their impact on the final properties of the material are still under debate. To illustrate this, it has to be first mentioned that it has been traditionally accepted that fluorination of porous carbons increases their hydrophobicity due to the repulsive nature of the fluorine atoms and/or the hindering of the water cluster formation (Li et al., 1995; Setoyama et al., 1996; Parmentier et al., 2012; Liu et al., 2019). However, recent simulation and experimental investigations on silicon carbide derived nanoporous carbons pointed toward a less straightforward and dual scenario (Farmahini et al., 2015; Shahtalebi et al., 2016). This two-fold behavior apparently arises from the fact that fluorination indeed generates more hydrophilic carbon surfaces but at the same time enhances the energy barriers for water sorption. The apparent discrepancies between these results may arise from the great number of variables involved. It has to be taken into account that besides the fluorination conditions (fluorine agent, temperature, reaction time…), the final chemical composition, as well as the structure of the products obtained by fluorination, are dependent on the structure and characteristics of the starting material (e.g. degree of graphitization, structural order, dimensions of the particles, surface curvature, purity…) (Lee et al., 2007; Matei Ghimbeu et al., 2015).

In this context, the present work aims at further investigating the role of fluorine doping on the experimental water sorption behavior of activated carbon fibers. To attain this goal, a pitch-based activated carbon fiber was submitted to a fluorination treatment under different conditions of partial pressure and temperature. The textural and surface properties of the so-obtained materials were characterized by means of gas sorption and XPS, while their water sorption behavior and stability was deeply investigated by single and consecutive water isotherms. The gathered results stress the importance of adequately choosing the fluorination treatment variables, since a small variation can lead to materials with quite different properties. Understanding how fluorine doping can variously change the features of ACFs is of particular importance in order to design materials with improved performances in function of the intended applications.

## Experimental

### Fluorination of the ACFs

Commercial pitch-based activated carbon fiber (Ad'all A-10, Osaka gas Co Ltd) was used as raw material and denoted as sample E. The surface fluorination of ACFs was performed by direct fluorination. This method has received a substantial attention because of its potential for uniform modification, short reaction time, low cost, and efficiency (Lee et al., 2007). The reaction between activated carbon fibers and fluorine is exothermic. To control the rate of fluorination and allow the heat of reaction to dissipate, F_2_-N_2_ gas mixture was used at low temperatures.

The fluorination device consisted of a reactor, a vacuum pump and a buffer tank connected to gas cylinders. The samples (around 400 mg) were loaded into a fluorine-passivated nickel reactor and degassed at 373 K for 1 h to remove moisture. Then diluted fluorine was introduced into the reactor at a very low flow. Nitrogen gas (99.999%) and fluorine gas (99.8%, Messer Grieheim GmbH) were used during the fluorination process. Fluorination was performed at room temperature and 1 bar for 10 min at three different F_2_:N_2_ gas volume ratios: 1:9, 3:7, and 5:5, thus obtaining ACFs A, B and C. After the reaction, the reaction vessel was then pumped out again to 10 mTorr with subsequent nitrogen gas purge prior to the extraction of the fibers. An additional sample (ACF D) was prepared introducing the F_2_-N_2_ mixture with a partial pressure of 3:7 and then heating the reactor to 150°C. The comparison of the results obtained for fibers B and D will allow to study the effect of the fluorination temperature on the water sorption behavior as well.

### Characterization of the ACFs

#### Nitrogen and Carbon Dioxide Sorption Isotherms

Nitrogen and carbon dioxide isotherms at −196 and 0°C, respectively, were performed in an Autosorb-1 device (Quantachrome Instruments). The N_2_ isotherms were used to calculate the BET specific surface area (S_BET_), micropore volume (V_micropore_, calculated by the Dubinin-Radushkevich (DR) equation) and total pore volume (V_t_ at p/p_0_ at 0.99). The volume of pores smaller than 0.7 nm (W_0_) was assessed from the CO_2_ adsorption isotherms with the DR equation. The samples were degassed overnight at 150°C prior to the sorption measurements. On this subject, several outgassing temperatures were investigated in order to find the optimal one (i.e., to guarantee a proper cleaning of the surface to allow the diffusion of the gas, while minimizing the modification of the original material). The extent of the influence of this variable was directly proportional to the degree of fluorination of the material.

#### X-Ray Photoelectron Spectroscopy (XPS)

A MultiLab 2000 spectrometer (Thermo Electron Corporation, UK) was used to identify the elements present in the external surface (<10 nm) of the fibers. Aluminum Kα (1485.6 eV) radiation was used as the X-ray source, and an anode voltage of 14.9 keV, a filament current of 4.6 A, and an emission current of a 20 mA were applied. All samples were treated at 10^−12^ bar to remove impurities. Survey spectra were obtained at a pass energy of 50 eV in increments of 0.5 eV.

#### Water Sorption

Water sorption isotherms were measured at 20°C using a gravimetric water sorption analyzer (Aquadyne DVS, Quantachrome Instruments). Each point of the gravimetric isotherm was obtained after an equilibrium time corresponding to 0.0004% of the mass change per minute at a given relative humidity. In order to check the stability of the materials after the exposure to humidity, cycling isotherm were also measured for all the materials. This means that after the first water adsorption-desorption cycle, the sample was kept in the analysis device and immediately submitted to at least one additional identical cycle. Since water is sensitive to the surface groups of the materials, mild outgassing conditions (100°C, overnight) were used. This guarantees an optimal compromise between the cleaning of the surface and avoiding the modification of the surface chemistry (Velasco et al., 2019).

## Results and Discussion

### Textural and Surface Properties of the ACFs

First, the modification of the surface chemistry of the carbon fibers by the fluorination treatment was evaluated by XPS. The XPS spectra revealed the presence of three main peaks, which correspond to F1s, O1s, and C1s. As can be seen in Table 1, the fluorine content increased as a function of partial pressure, ranging from 12 to 30 at. % (18 to 39 wt. %). The surface treatment slightly affected the content of oxygen, showing a meager increase for ACF A, followed by a gradual decrease with the raise of the F_2_:N_2_ ratio. In this regard, some C-H and C-C bonds can be broken during the fluorination process, forming dangling bonds, which are likely to subsequently react with oxygen and moisture from air and form oxygenated surface groups (Dubois et al., 2018). The presence of oxygenated groups in the starting material may also affect the nature and stability of the C–F bonding (Matei Ghimbeu et al., 2015). On the other hand, samples B and D exhibit a similar surface composition, thus indicating a low impact of the temperature in the extent of the fluorination.

**Table 1:** Surface elemental composition of the ACFs obtained from the XPS spectra.

**Sample**	**Elemental contents (at. %)**		
	**C**	**O**	**F**
E (raw)	90.1	9.9	–
A (1:9)	75.9	11.4	12.7
B (3:7)	65.8	10.4	23.8
C (5:5)	60.5	9.8	29.7
D (3:7, 150°C)	68.5	9.8	21.7

**Figure 1:**
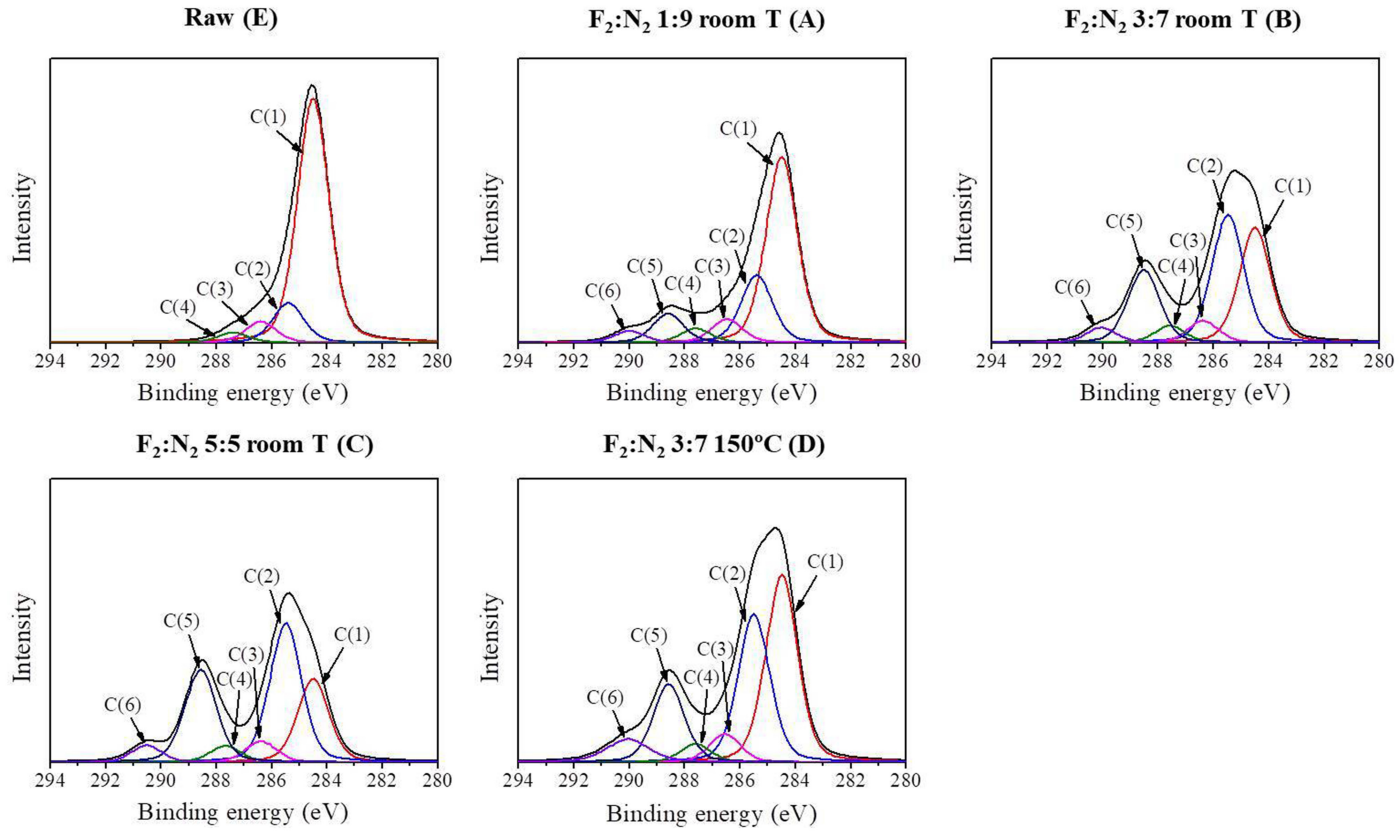
Deconvolution of the XPS C1s spectra of the ACFs.

In order to evaluate the changes in the surface functional groups of the fluorinated ACFs, the C1s peaks were deconvoluted to several pseudo-Voigt functions using a peak analysis program obtained from Unipress Co, USA. More details can be found in previous works (Kim et al., 2019). The assignments and peak parameters of the different C1s components are provided in Table 2 and the deconvolution of the C1s spectra in Figure 1. As expected, it is observed that fluorination changes the valence state of carbon bonding from sp^2^ to sp^3^. Also, the semi-covalent C-F bonding markedly increases with the fluorine content, following the order A < B < C. These C-F bonds are essentially covalent and can result from the hyperconjugation between C–C bonds in the non-fluorinated region and closed to C–F bonds (Sato et al., 2004). On the contrary, the different results obtained for B and D samples indicate that the formation of covalent bonding is favored by an increase of the treatment temperature. These results are in agreement with the literature (Bismarck et al., 1997; Kim et al., 2019).

**Table 2:** Parameters obtained from the deconvolution of the C1s peak of the XPS spectra.

**Components**	**Peak position (eV)**	**Concentration (at. %)**				
		**E (raw)**	**A**	**B**	**C**	**D**
C(1) C-C(sp2)	284.5	77.3	55.93	31.04	22.57	37.88
C(2) C-C(sp3)	285.5	12.68	20.38	34.54	37.05	29.88
C(3) C-O	286.4	6.75	7.11	6.01	5.68	5.81
C(4) C=O	287.5	3.27	4.26	4.63	4.54	3.71
C(5) Semi-covalent C-F	288.6	–	8.77	19.65	25.08	15.80
C(6) Covalent C-F	290.1	–	3.55	4.13	4.63	6.92

**Figure 2:**
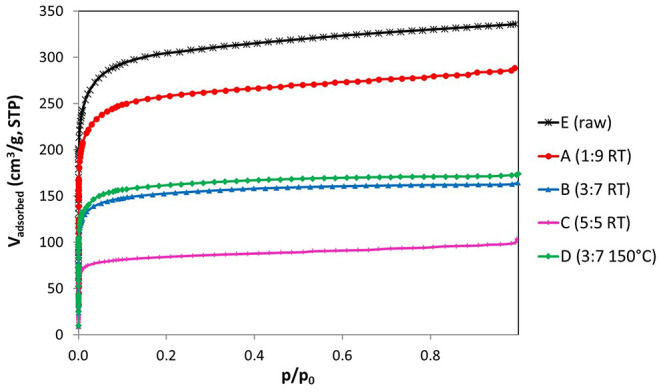
N_2_ adsorption isotherms at −196°C of the ACFs outgassed at 150°C.

The nitrogen adsorption isotherms of the ACFs (Figure 2) display important information about the impact of the fluorination treatment on the textural properties. The parent fiber (E) exhibits a type I isotherm characteristic of microporous materials. It is noticed that the increase of the fluorine content is accompanied by a progressive reduction of the microporous structure (Table 3). In this sense, while a moderate fluorine doping (ACF A) barely etches the pore walls, thus largely preserving the textural properties, more severe conditions can reduce several times the nitrogen uptake (ACF C). However, the shape of the isotherm is preserved for all the fluorinated fibers indicating only a reduction of the micropore volume available due to the formation of the C-F bonds. This is further confirmed by the calculation of the average pore width by the QSDFT-slit kernel, which only shrinks from 0.61 nm for the initial sample to 0.57 nm for the fluorinated ones and it is in accordance with previous works (Lee et al., 2007). Besides, the specific density of the carbon increases due to the weight uptake after the reaction with F_2_. This means that the net reduction in the micropore volume due to the fluorination is actually smaller than the measured one (Setoyama et al., 1996; Parmentier et al., 2012). The volume of narrow micropores calculated from the CO_2_ isotherms (Table 3) confirms the previous statements.

**Table 3:** Textural parameters obtained from the N_2_ and CO_2_ adsorption isotherms at −196 and 0°C, respectively.

	**N**_****2****_			**CO_**2**_**
**Sample**	**V_**micropores**_ (cm^**3**^/g)**	**V_**t**_ (cm^**3**^/g)**	**S_**BET**_ (m^**2**^/g)**	**W_**0**_ (cm^**3**^/g)**
E	0.44	0.54	1,194	0.325
A	0.38	0.45	1,011	0.286
B	0.24	0.25	600	0.181
C	0.14	0.15	331	0.105
D	0.25	0.27	606	0.189

### Water Sorption Behavior

**Figure 3:**
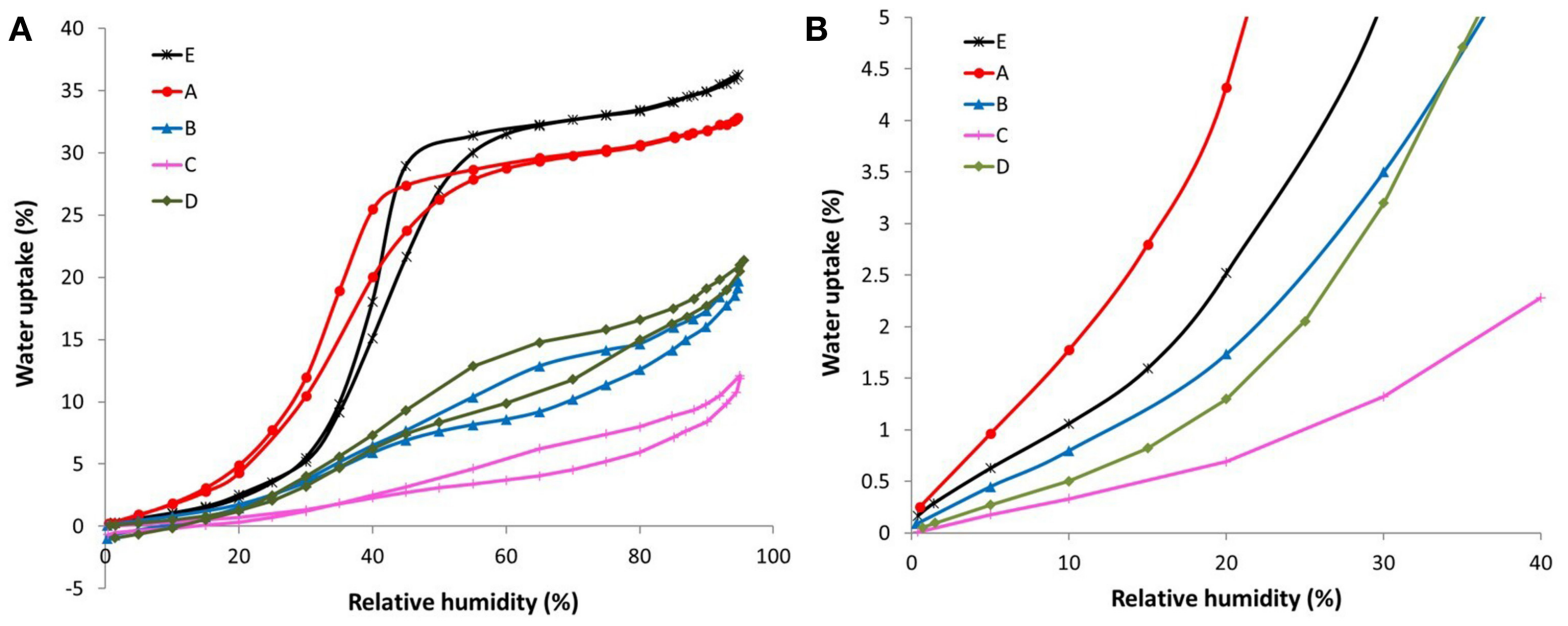
**(A)** Complete and **(B)** low pressure range of the H_2_O sorption isotherms at 20°C of the ACFs.

The water sorption isotherms of all the ACFs herein investigated are displayed in Figure 3A, together with a magnification of the adsorption branch at low relative humidities (Figure 3B). Therein it is observed that the fluorinated samples exhibit a quite different water sorption behavior depending on their degree of fluorination. On the one hand, the water isotherm of sample A, with the lowest fluorine content, is quite similar to the one of the raw activated carbon fiber E. This type V isotherm, with a marked upswing in the water uptake at medium relative humidities followed by a more steady profile, is characteristic of microporous materials. Still, some non-negligible differences are noted: sample A shows (i) a higher water affinity at low relative humidities (see Figure 3B), (ii) a less pronounced slope of the upward curve, and (iii) a slightly lower total water uptake. Considering that the water adsorption process is initially governed by the specific interactions between the water molecules and the active sites on the carbon surface, the first point denotes an increased hydrophilicity of the material inferred by the mild fluorination. The formation of some additional oxygen groups during or after the fluorination can contribute to some extent to this enhanced water affinity, although it cannot justify such a notorious change. In this regard, the Langmuir-type equation developed by Lodewyckx et al. (2013) permits calculating the amount of oxygenated complexes of porous carbon materials by fitting the first points of the water adsorption isotherm, assuming these are the only type of surface groups playing a role. By applying this model, a value of 19.9% wt. is obtained for sample A, far from the XPS result (13.6% O wt.) (Table 4). Thus, it seems that this increased hydrophilicity can be mainly ascribed to the presence of the fluorine atoms. The second point indicates a slower filling of the micropores, probably due to increased internal barriers for water diffusion in the fluorinated system (Farmahini et al., 2015). However, it has to be noted that these barriers can only delay but not hamper the sorption of water, since the drop in the total water uptake after the mild fluorination is only of around 3% (point iii). As a matter of fact, this is the only doped fiber that presents a higher ratio of pore volume obtained from water vapor adsorption to total pore volume measured by nitrogen than the original fiber E.

**Table 4:** Comparison between the XPS oxygen contents and the ones calculated from the water sorption isotherm (% weight).

	**[O]_**XPS**_**	**[O]**_****waterisotherm****_	
		**1st water cycle**	**2nd water cycle**
E	12.8	13.0	**—**
A	13.6	19.9	21.4
B	11.8	12.1	13.5
C	10.8	8.3	9.6
D	11.2	10.7	13.8

Similar water isotherms were gathered for samples B and D but following a different pattern than that of ACFs A and E. In this case, the most noticeable water uptake occurs at high relative pressures (>0.65), preceded by an initial rise and a subsequent plateau. In accordance with the textural data, sample D leads to a slight higher total water uptake. Though, an opposite trend is observed at low relative humidities (Figure 3B). Bearing in mind their similar fluorine and oxygen contents, this seems to be related to the variances in the amount of covalent and semicovalent C-F bonds in each material. In particular, sample D has more covalent C-F bonds than B (6.92 vs. 4.13%, respectively) and less semi-covalent (15.8 vs. 19.7%). It has already been established that larger dipoles of the ionic or semi-covalent C-F bond provide greater polarity than that of the covalent C-F bond (Nakajima, 1994). Also, the influence of the probable presence of hydrophobic –CF2 of –CF3 bonds cannot be disregarded.

In line with the textural parameters calculated from the nitrogen isotherms, sample C, with the highest extent of fluorination, shows the lowest capacity for water adsorption at all the relative vapor pressures.

According to Table 4, if the model based on the only influence of oxygen is applied again, the oxygen content calculated from the water isotherm for sample C is lower than the XPS value, suggesting an increased hydrophobicity. However, there are other variables that could play a role. For example, steric effects, as those reported for highly oxygenated carbon materials (Velasco et al., 2016), could exist, leading to a decrease of the number of water molecules in the cluster or even avoiding the formation of the cluster. In the case of samples B and D, both the calculated and the experimental amount of oxygenated complexes are similar, which can lead to the misleading conclusion that the fluorine is not participating in the water affinity of these samples. Nevertheless, a very probable scenario would be that the two effects identified for fibers A (hydrophilicity given by the fluorine) and C (steric hindrance) are counteracting each other. This somehow intermediate performance is in line with the in-between fluorine content of samples B and D with respect to A and C fibers. Summing up, the trend in calculated oxygen against real oxygen for all samples evidences the supplementary influence of the fluorine on the water adsorption.

On the other hand, the desorption branch of the water isotherms of these three samples goes to slightly negative values. This indicates some mass loss. It is highly probable that some labile C-F bonds are present in the surface of the materials, especially in the case of fluorination at low temperatures and during short times, and react with the water molecules to form hydroxyl groups (Parmentier et al., 2012). This could also explain the uptake of water at high relative humidities of these samples.

#### Water Cycles

**Figure 4:**
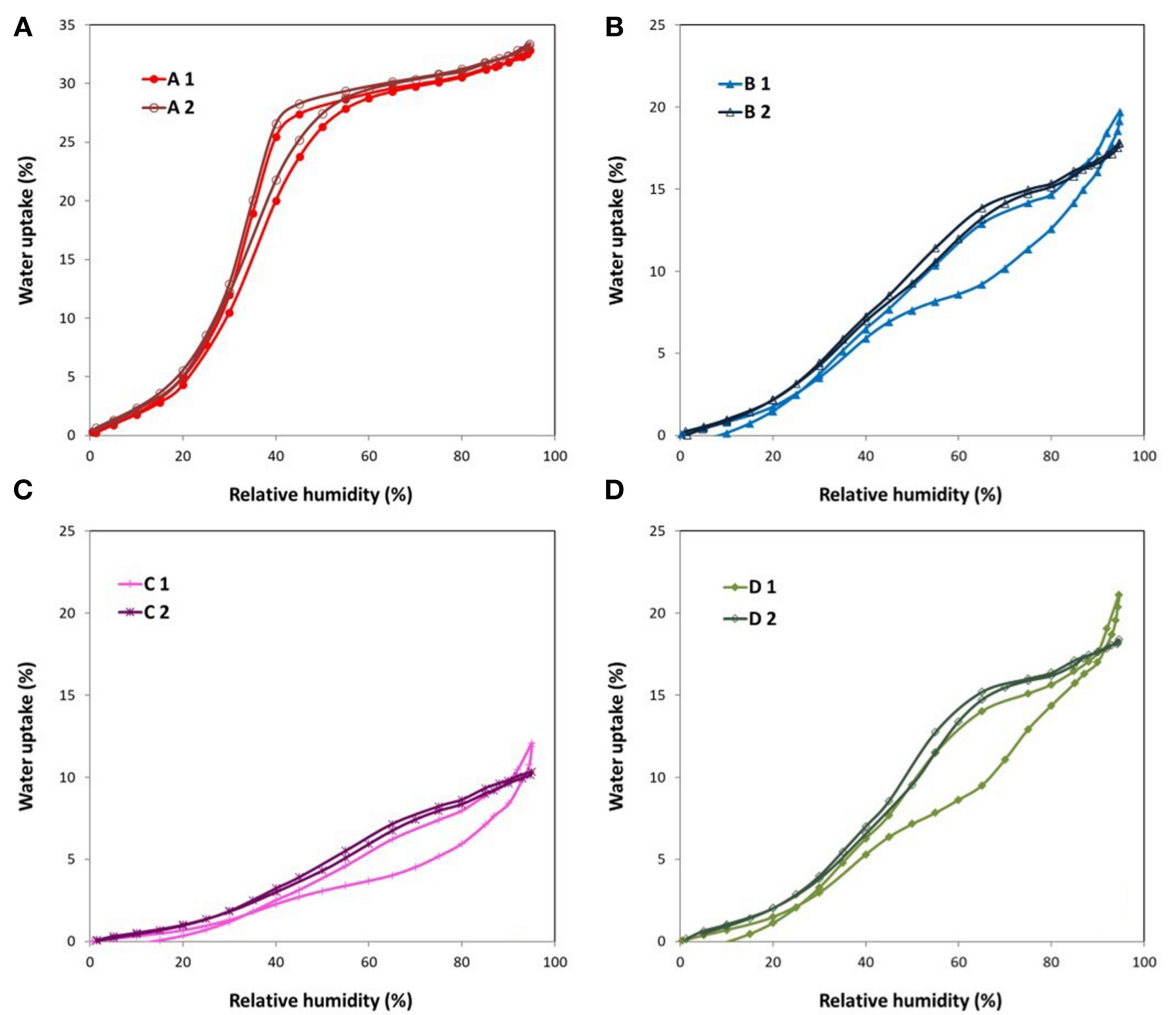
Water cycling isotherms of the fluorinated ACFs **(A)** A, **(B)** B, **(C)** C, and **(D)** D.

In order to further investigate and to check the stability of the fluorine doped ACFs under humid conditions, consecutive water isotherms were measured. The results are presented in Figure 4. Therein a slight increase in the water uptake of sample A is noticed, most probably due to a minor oxygenation of the surface (see Table 4). On the contrary, the rest of the fibers show a much more marked change of the adsorption profile. More in detail, the hysteresis loops nearly disappear in the second cycle and the adsorption/desorption branches practically overlap with the adsorption branch of the initial isotherm. Furthermore, there is a slight decrease of the total water uptake in the second cycle, due to the flattening of the adsorption curve at very high relative humidities. This could confirm our previous hypothesis of the reaction of the water molecules with the labile C-F bonds at very high vapor pressures during the first cycle. In the second cycle, no labile C-F bonds were left for reaction with water. Also, there is a non-negligible raise in the oxygenated species calculated from the water isotherms (Table 4) between the first and the second cycle. Since no changes were recorded in the nitrogen isotherm of these fibers after the first water cycle, this shift in the profile of the water isotherms seem to be only caused by alterations of the surface chemistry that facilitate the penetration of the water molecules in the porous network.

An additional 3rd water cycle was performed on the highly fluorinated samples B, C, and D, obtaining essentially the same isotherms as those of the 2nd cycle. This will be further discussed in the next section.

#### Kinetics

Water vapor adsorption kinetics in porous carbon materials are dependent on both the pore size and the functionality of the carbon surface (Fletcher et al., 2007). Since the fluorine-doped fibers present the same average pore width, any differences in the kinetic profiles need to be assessed in terms of the surface chemistry.

**Figure 5:**
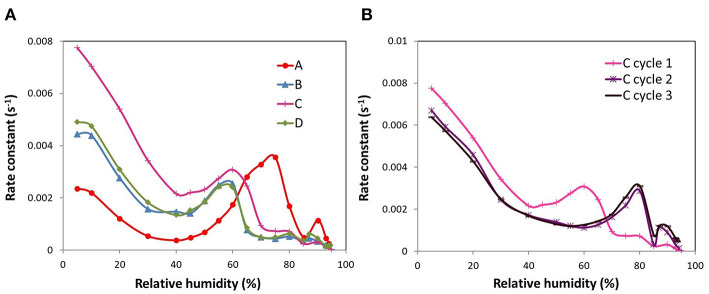
Rate constants of the ACFs as a function of relative humidity: **(A)** fluorinated ACFs and **(B)** first to third cycle for ACF C.

The Linear Driving Force (LDF) model was used in order to study the kinetics of water adsorption in the ACFs (*R*^2^ > 0.99). The kinetic profiles thus estimated are shown in Figure 5A. For ACF A, a typical profile of water adsorption in microporous pitch-based activated carbon fibers is obtained (Ito et al., 2015). This implies that the rate constant of water adsorption initially decreases with increasing water vapor pressure until it reaches a minimum, which is coincident with the steepest water uptake, then raises again as the isotherm is getting flatter and there is a sharp decrease at high relative humidities as a result of a small change in the slope of the isotherm. Thus, the rate-determining step is related to the formation of water molecular assemblies. This agrees with previous works where it was found that slower kinetic rates are assigned to associative hydrogen bonding of water molecules to adsorbed water (Fletcher et al., 2007). For the other fluorinated ACFs the first minimum is reached approximately at the same relative pressure than for sample A but with a less marked character. Then, the subsequent kinetic profile is displaced to lower relative humidities, so that the amplitude of the peak matches with the plateau of the water sorption curve, and then there is a fall of the adsorption rate to the actual minimum. Another interesting observation is that the kinetic rates at the first stages of the water sorption process, where the surface chemistry plays the most important role, follow the order A < B ≤ D < C. Taking into consideration that the adsorption kinetics are slower when the adsorbate-adsorbent interactions are stronger (Fletcher et al., 2007; Jia et al., 2017), this corroborates our previous findings about the hydrophilic/hydrophobic nature of the studied ACFs: while the mild fluorinated ACF (A) presents a marked hydrophilic performance, the one with the highest fluorine surface coverage (C) leads to the weakest surface interactions, thus to a hydrophobic behavior.

Finally, the kinetic profiles of the consecutive water adsorption cycles of the ACF with the highest fluorine content (C) were also compared (Figure 5B). The changes in the surface chemistry produced by the exposure of the sample to one water sorption cycle, varied the kinetics of the adsorption process as well. In fact, the kinetic profile of cycles 2 and 3 resembles that of sample A, typical of a continuous micropore filling. Moreover, the adsorption rate at low relative humidities slows down after the first cycle, supporting again the formation of surface groups with higher water affinity. The same trends were also found for fibers B and D.

Summing up, the obtained results highlight the importance of controlling the fluorination process in order to obtain porous carbon materials with the desired textural and surface features, in function of the properties of the starting material (pore width, particle size, graphitization level…) and the intended application. This will be crucial in order to guarantee an optimal efficiency and stability of the samples under humid conditions.

## Conclusions

In this work, a series of fluorine-doped pitch-based activated carbon fibers were obtained by gradually changing the partial pressure of the gas and to a minor extent, the temperature. This led to samples with varied fluorine content, C-F type bonding and textural properties, as revealed by the information given by XPS and gas sorption isotherms. Also, water isotherms with different profiles were collected.

A mild fluorination (F_2_:N_2_=1:9, room temperature) slightly modified the textural properties of this particular parent fiber and inferred hydrophilic properties to the carbon surface. This was corroborated by a marked increase in the slope of the water isotherm at low relative pressures accompanied by slower kinetics, due to strong adsorbate-adsorbate interactions, in comparison with the rest of the samples. Differently, the high fluorine surface coverages obtained by using elevated fluorine gas ratios led to a decreased porosity and an increasing hydrophobic surface. In addition, the formation of labile C-F bonds was promoted and this caused a certain instability of the materials when exposed to water vapor. Raising the fluorination temperature up to 150°C did not have an apparent influence on the total fluorine content. However, these 2 samples exhibited a somehow different sorption behavior that can be attributed to the different nature and siting of the C-F bonds formed. These findings put into evidence that even slight changes in the reaction conditions can be decisive in the modulation of the properties of the final fluorine decorated material.

Summing up, water vapor sorption has revealed as a useful technique for the characterization of the non-straight forward porous and surface modifications induced by fluorine doping of carbon materials. The provided information will certainly contribute to widen and improve the potential applications of fluorinated activated carbon fibers.

## Data Availability Statement

The raw data supporting the conclusions of this article will be made available by the authors, without undue reservation.

## Author Contributions

LV studied the water sorption behavior of the samples and wrote the manuscript. KK synthesized and characterized the raw and fluorinated activated carbon fibers. Y-SL and PL conceived the work and contributed to the discussion of the results. All authors contributed to the article and approved the submitted version.

## Conflict of Interest

The authors declare that the research was conducted in the absence of any commercial or financial relationships that could be construed as a potential conflict of interest.
